# Meaning Matters: A Person-Centered Investigation of Meaning in Life, Future Time Perspective, and Well-Being in Young Adults

**DOI:** 10.17505/jpor.2024.27189

**Published:** 2024-12-13

**Authors:** Luigia Simona Sica, Anna Parola, Barbara De Rosa, Massimiliano Sommantico, Elisabetta Fenizia, Jacopo Postiglione, Giorgio Maria Regnoli, Santa Parrello

**Affiliations:** Department of Humanities, University of Naples Federico II, Naples, Italy

**Keywords:** Meaning in life, optimism, future, person-centered approach, perspective coping

## Abstract

Within the framework of positive psychology, this study aims to investigate whether meaning in life, optimism and future orientation have acted together as a psychological resource in coping with a non-normative challenge such as the Covid-19 pandemic. 389 respondents participated in this study. Future time perspective, presence/search for meaning in life, life orientation and dimensions of well-being (anxiety, depression, stress and aggressive behavior) were assessed. A person-centered approach through latent profile analysis (stepwise approach) was employed. In addition, multinomial logistic regression was used to investigate whether gender, age group, student/employment status and loss episodes during the pandemic predicted profile membership. Latent profile analysis identified three profiles: Aggressive coping (Profile 1, 30%, n = 117), Perspective coping (Profile 2, 29%, n = 114), and Flattened coping (Profile 3, 41%, n = 158). The results support the hypothesis that the presence of meaning in life, a positive life orientation and a positive view of the future act as coping strategies against stressful situations. Practical implications for supporting these resources in young people are discussed.

## Introduction

Understanding what factors determine an individual's psychological well-being is one of the central tasks of positive psychology. Understanding what personal characteristics enable individuals, especially young people, to cope with critical events and move toward positive self-development is a specific task of positive developmental psychology. On the other hand, the developmental perspective allows us to address the issue of individual well-being and adaptation to context in a complex manner and at the same time allows us to identify, from a prevention and intervention perspective, those characteristics (personal, contextual, social) that enable each individual to proceed toward positive rather than negative developmental trajectories.

From this perspective, identifying the psychological elements that characterize individuals who are best able to adapt to the environment, who define their identity in an optimal way, and who can proceed along the developmental path using moments of crisis as developmental challenges is a central topic of developmental psychological research. This is especially true when we consider that during each person's life cycle, the events that arise as tasks to be faced are not always normative in nature (i.e., they are not always predictable and, as such, addressable with prior knowledge and experience), but often unpredictable and unexpected, as well as new. In this case, understanding which psychological characteristics enable individuals to cope successfully with such challenges (and which do not) is an important and crucial step in psychological intervention especially with young people.

With this in mind, positive psychology has extensively studied a number of characteristics (such as, resilience, happiness, strengths; Seligman & Csikszentmihalyi, 2014) that support individuals in individual well-being. Among them, meaning in life, optimism and future orientation are psychological skills for well-being in a positive psychology perspective. Meaning in life is an element related to the pursuit of eudemonia and can help to achieve happiness and well-being (Lambert D’raven & Pasha-Zaidi, 2016). In the same way, optimism and future orientation are generally regarded as critical for well-being, motivation and behaviour (Kooij et al., 2018).

In order to understand whether these elements can be resources to face the developmental challenges during the life span, and thus, represent psychological dimensions to be supported and empowered in young people to support them in their positive development, we asked whether they act as resources even in situations of difficulties that are not preventable or predicted, i.e., not normative. The recent crisis related to the Covid-19 pandemic represents in this sense a privileged moment of investigation to answer our question, since it represented an unexpected event, a historical challenge, that changed (at least for a defined period) the daily life of each of us.

Hence, the objective of our study is to investigate if meaning in life, optimism and future orientation have acted together as psychological resources to cope with non-normative challenges (namely, Covid-19 pandemic), to support the hypothesis that these resources could define a typology of "coping strategies" that can be supported in young people to face developmental challenges.

### Meaning in Life, Time Perspective and Optimism: Why them?

Studies show a strong relationship between meaning in life and well-being (Ho et al., 2010, McMahan & Renken, 2011). Among healthy psychological functioning, more meaning in life has been related to life satisfaction (Joshanloo, 2019) and happiness (Li et al., 2019). Furthermore, meaning in life (MIL) is positively related to psychological well-being across almost every stage of the life span (King et al., 2006; Reker et al., 1987; Zika & Chamberlain, 1992) and it plays a significant role in both personal and vocational optimal development (Parola et al, 2022). A growing body of literature has indicated its adaptive function, such as promoting mental as well as physical health (Hooker et al., 2018; King & Hicks, 2021).

As stated by Steger, meaning in life “enables people to interpret and organize their experience, achieve a firm sense of their own worth and place, identify the things that matter to them, and effectively direct their energies” (2009, p. 680).

Meaning in life encompasses the perception of order, coherence, and significance in one’s existence, along with the pursuit and realization of worthwhile objectives, leading to a sense of fulfillment (Reker, 2007). However, a pivotal aspect in understanding meaning in life is the concept of the will to meaning (Frankl, 1963), as its absence can result in psychological distress. Steger and colleagues (2006) proposed a definition of meaning in life as the interpretation and importance one assigns to the essence of their being and existence. Within this framework, two distinct dimensions of meaning in life are delineated: “presence of meaning” and “search for meaning.” *Presence* of meaning refers to the degree to which an individual perceives or experiences meaning in their life, while the *search* for meaning pertains to the extent of one’s quest for significance in life. These facets are not mutually exclusive: individuals devoid of meaning may actively seek it, and those who already perceive meaning may still explore additional or alternative sources of significance (Steger et al., 2011).

In the context of the COVID-19 pandemic, psychological resources, such as positive perception of the future, finding meaning in life and optimism may be extremely important for coping with the difficult and new situation and for maintaining psychological well-being (Lasota & Mróz, 2021). Among them, positive psychology indicates Time perspective (TP) as a central aspect of human daily psychological functioning. Positive future orientation is strongly associated with a range of various mental well-being indicators (Burzynska & Stolarski, 2020). Loose and colleagues (2021, 2022) asked how dispositional temporal perspectives might have affected college students’ ability to cope with the COVID-19 pandemic while preserving their well-being, but also whether the pandemic experience was powerful enough to change their temporal perspective. Indeed, research has shown that future orientation – understood as a positive future time perspective – decreased in the population after the 9/11 terrorist attacks (Holman et al., 2005; Holman et al., 2016), as well as in Israeli and Palestinian adolescents exposed to war events of traumatic magnitude (Solomon et al., 2005; Seginer & Schlesinger, 1998). In the study by Loose and Colleagues (2022), the pandemic did not appear to have had similar effects, perhaps because, as the authors comment, it was carried out at a stage that was not particularly dramatic. Indeed, 60% of the students involved reported thinking more about the future since the beginning of COVID-19, 40% about the present, and 22% about the past, showing more psychological distress and learning difficulties in the latter case.

Psychological time has garnered interdisciplinary interest for over five centuries (Stolarski et al., 2018), with contemporary frameworks highlighting three interdependent dimensions of time experience (Wittmann and Lehnhoff, 2005): (1) time estimation abilities, assessed through clock time accuracy, (2) time awareness, reflecting subjective perceptions of time’s pace (fast or slow), and (3) time perspective, encompassing individuals’ unconscious personal orientations towards time, organizing the continuous flow of existence into meaningful categories (Zimbardo and Boyd, 2008). Time perspective involves (a) time attitudes, representing emotional evaluations of the past, present, and future (Mello et al., 2016), and (b) temporal focus, indicating the cognitive investment in past, present, or future considerations (Shipp et al., 2008). While perceptions of time’s speed vary with circumstances (Rutrecht et al., 2021; Witowska et al., 2020a, 2020b), individuals allocate differing amounts of time to temporal reflections based on context (Holman and Silver, 2006). Temporal focus (TP), as conceptualized by Zimbardo’s theory (Holman & Zimbardo, 2009), denotes the unconscious cognitive construction of life experiences, influenced by temporal orientation across past, present, and future. Temporal future focus (FTF) signifies the attention individuals dedicate to future considerations, whether positive or negative (Shipp et al., 2008).

One among the resources identified by positive psychology as promoting personal well-being is certainly optimism. It is, among other things, closely linked to the temporal perspective as described so far since its definition underlines that link: “Optimism describes a positive orientation towards the future. Optimists are people who have the habitual tendency to expect positive future outcomes even when difficulties arise” (Scheier & Carver, 1992). Or, more recently, it has been defined as “the extent to which people hold generalized favourable expectancies for their future” (Carver et al. 2010, p. 879).

Scheier and Carver’s (1985) self-regulatory model of dispositional optimism stands as the primary theoretical framework for comprehending optimism, framing human behavior within the context of goal regulation. Drawing from expectancy-value models of motivation (Atkinson, 1964), they pinpoint two pivotal motivators for goal pursuit: value, reflecting the subjective significance of a goal, and expectancy, representing one's confidence in achieving it. Optimists exhibit a consistent inclination towards anticipating positive outcomes, cultivating a pervasive sense of confidence that propels them towards achieving their goals, even amidst adversities, thereby fostering heightened overall dedication. Conversely, pessimists typically harbor more skepticism, leading to disengagement when confronted with obstacles to goal attainment. A recent study (Miao et al., 2021) has explored the future-oriented function of meaning in life. The authors argued two important points: the first, that MIL promotes hope through the mediating role of FTF; the second, that “MIL’s future oriented function is reflected in the contexts of both everyday life and coping with adversity” (Miao et al., 2021, p. 5).

It is precisely this last point, coping with adversity, which is most poorly explored, and which is the specific object of the present study. The general objective of the present study is, in fact, precisely to understand whether young people with a positive disposition towards the future supported by the presence of meaning in life have maintained adequate well-being even during a particularly difficult situation, such as that of the pandemic experience.

### The Current Study

In the current study of future time perspective and well-being in young Italian adults during the COVID-19 pandemic, we have assumed that time perspective, optimism and meaning in life are potentially crucial elements in the resolution of young people’s psycho-social challenges. We examine whether time perspective could be connected to optimism and meaning in life in defining specific individual modalities of coping with difficult situations (i.e., the covid pandemic). They may have helped to counteract widespread pessimism (Wu, 2022) and feelings of meaninglessness (Buccolo et al., 2020) during the pandemic period, protecting against internalizing and externalizing disorders. In order to explore this goal, we used a person-centered analysis to identity different types of people in terms of profiles of time perspective, optimism, meaning in life and well-being/malaise (which from now on we will define as types of perspective coping). We had two broad research questions:

*Research question 1*: How many distinct profiles of time perspective, optimism, meaning of life and well-being would emerge, in terms of within-profile homogeneity and between-profile heterogeneity? We assumed that profiles with the highest levels of future orientation in coping with problematic situations, and of optimism and meaning in life, would show the lowest levels of negative psychological functioning.

*Research question 2*: What is the effect on profile membership of gender, age group, student/employment status, and loss episodes during the pandemic and economic situation after the pandemic? No specific assumptions were made about gender, age group, student/employment status. But we hypothesized that individuals’ post-covid economic situation and episodes of loss during the pandemic would predict the profile with the highest scores on the negative dimensions, in line with the cited covid literature.

## Method

### Procedure and Participants

Participants were enrolled online through advertisements on social media (e.g., Facebook) between April 1, 2021, and May 31, 2021. Data were collected via self-report questionnaires administered through an Internet-based survey and subsequent snowball sampling: recruited participants were prompted to identify additional potential respondents within their social circles, who were, in turn, then invited to nominate others from their own social networks, continuing the chain. Participants were considered eligible for participation if they met the following inclusion criteria: (a) between 18 and 34 years old and (b) compliant with the quarantine/isolation measures. It was ensured that participants in the research remained anonymous and participated voluntarily, with the ability to discontinue questionnaire completion at any point.

389 respondents (73.5% females; *M* = 23.5, *SD* = 4.4) took part in this study. 54.2% of the participants had completed secondary school, and 45.7% had completed a university degree. Most participants (78.1%) were students, and 31.4% were employed.

### Measures

#### Socio-demographic information

Respondents provided socio-demographic data, i.e., age, gender, region of residence, level of education, and occupational status (student/employee). They were also asked to report information about their condition during the COVID-19 pandemic, such as whether their family had suffered economic damages due to the pandemic and whether they knew people who had died of COVID-19.

#### Future Time Perspective Scale for Adolescents and Young Adults (FTPS-AYA; Lyu & Huang, 2016)

*The Future Time Perspective Scale for Adolescents and Young Adults* (FTPS-AYA; Lyu & Huang, 2016) was used to assess the future time perspective. This measure is composed of 28 items divided into six scales: Future Negative (7 items), Future Positive (5 items), Future Confusion (4 items), Future Perseverant (5 items), Future Perspicuity (3 items), and Future Planning (4 items). Future Negative is a view of the future characterized by fear, anxiety and hopelessness. Future Positive is a vision of the future characterized by hope for success and optimism. Future Confusion refers to a vision of the future characterized by uncertainty and lack of clarity. Future Perseverant is the belief that one must work hard to overcome failure and adversity. Future Perspicuity refers to a clear vision of the future. Future Planning relates to goal setting and commitment to future rewards.

Participants are asked to respond according to a five-point Likert-type scale ranging from 1 (= “strongly disagree”) to 5 (= “strongly agree”). Examples of items are: “I believe I am able to control my future through my own efforts” and “I move forward every day without making plans”. Mean scores on the items of each scale are calculated. In the present study, Cronbach *α* ranged from 0.571 (Future Positive) to 0.918 (Future Negative).

#### Depression Anxiety Stress Scale (DASS-21; Lovibond & Lovibond, 1995; Bottesi et al., 2015)

*The Depression Anxiety Stress Scale-21* (DASS-21; Lovibond & Lovibond, 1995; Italian adaptation and validation by Bottesi et al., 2015) was used to assess the distress dimension. This measure is composed of 21 items divided into three scales: Depression (7 items), Anxiety (7 items), and Stress (7 items). Participants are asked to rate the frequency and severity of depression, anxiety, and stress symptoms on a four-point Likert-type scale ranging from 0 = (“did not apply to me at all”) to 3 (= “applied to me very much, or most of the time”). Examples of items are: “In the last 7 days, I had difficulty relaxing” and “There was nothing to give me enthusiasm”. The scores on the items of each scale are summarized. In the present study, Cronbach’s *α* was 0.91 for Depression, 0.87 for Anxiety, and 0.88 for Stress.

#### Meaning in Life Questionnaire (MLQ; Steger et al., 2006; Di Fabio, 2014)

*The Meaning in Life Questionnaire* (MLQ; Steger et al., 2006; Italian adaptation and validation by Di Fabio, 2014) was used to assess the presence and search for meaning in life. This measure is composed of 10 items divided into two subscales: Presence of meaning (5 items) and Search of meaning (5 items). Participants are asked to respond according to a seven-point Likert-type scale ranging from 1 (= “Absolutely true”) to 7 (= “Absolutely untrue”). Examples of items are: “I am aware of what makes my life meaningful” and “I am always looking for something to make my life meaningful.” The scores on the items of each subcale are summarized. In the present study, Cronbach’s *α* was 0.86 for the Presence of meaning and 0.88 for the Search for meaning.

#### Aggression Questionnaire (AQ; Buss & Perry, 1992; Sommantico et al. 2008)

*The Aggression Questionnaire* (AQ; Buss & Perry, 1992; Italian adaptation and validation by Sommantico et al., 2008) was used to assess aggressive behaviour. This measure is composed of 29 items and four subscales: Physical Aggression (9 items), Verbal Aggression (5 items), Anger (7 items), and Hostility (8 items). Participants are asked to respond according to a 5-point Likert-type scale (ranging from 1 (= “Extremely uncharacteristic of me”) to 5 (= “Extremely characteristic of me”). Examples of items are: “I often feel like a barrel of gunpowder ready to explode” and “I do not hesitate to resort to violence to defend my rights.” Italian validation supports using a single factor (Sommantico et al., 2015) that is obtained by calculating the mean score on all items. In the present study, Cronbach’s α was 0.87.

#### Life Orientation Test-Revised (LOT-R; Scheier & Carver, 1985; Giannini et al., 2008)

*The Life Orientation Test-Revised* (LOT-R; Scheier & Carver, 1985; Italian adaptation and validation by Giannini et al., 2008) was used to assess dispositional optimism. This measure is composed of 10 items. Participants are asked to respond according to a five-point Likert-type scale ranging from 0 (= “Strongly disagree”) to 5 (= “Strongly agree”). Examples of items are: “I hardly believe that things are going in my favor” and “I am always optimistic about my future.” The scores on the items are summarized. In the present study, Cronbach’s *α* was 0.78.

#### Pandemic, Time, and Future Scale (PTFS; Parrello et al., submitted)

*The Pandemic, Time, and Future Scale* (PTFS; Parrello et al., submitted) assessed the situational time perspective. This measure is composed of 7 items that assess whether the experience of the pandemic has hurt the organization of time and vision of the future. Participants are asked to respond according to a five-point Likert-type scale ranging from 1 (= “Completely disagree”) to 5 = (“Completely agree”). Examples of items are: “This pandemic has changed me in a negative way” and “Compared to before the pandemic, I feel I have wasted time in achieving my goals”. In the present study, Cronbach’s *α* was 0.85.

### Data Analyses Plan

Preliminary analyses (means, standard deviations, skewness and kurtosis) were performed. Bivariate correlations were *computed* between all variables of interest. The MCAR test (Little, 1988) was used to assess the assumption of the missing at random for missing values. Results suggested data was missing completely at random (*χ2* = 6.177, *df* = 13, *p* =0.939). Thus, missing data were handled in the analysis using the full-information maximum-likelihood method (FIML) (Little & Rubin, 1989).

To identify the profiles, latent profile analysis (LPA) was performed on all participants, using time perspective, meaning in life, optimism, and well-being/malaise variables. LPA is a robust mixture-model technique commonly used to identify subtypes of homogeneous latent classes or subgroups within a large heterogeneous group (Garrett & Zeger, 2000; Hagenaars & McCutcheon, 2002). This iterative process clusters similar response profiles to create subgroups/classes. In this way, individuals were assigned to their most likely type based on their profile.

A stepwise approach was followed to determine the optimal number of profiles that best capture the data and sample, starting with two profiles and increasing the number of latent classes incrementally (Nylund et al., 2007). Therefore, the number or size of latent profiles was unknown and underestimated a priori. It was assumed that each individual belonged to one of a set of *n* latent profiles. The number was incremented until convergence problems or model fit indicated unlikely results.

In each step, fit information criteria, parsimony of classes and entropy statistics were examined. The Akaike information criterion (AIC; Akaike, 1987) and the Sample-sizeadjusted Bayesian information criterion (BIC; Schwarz, 1978) were used for the model fit. Good fit models are indicated by lower values of AIC and BIC (Feldman et al., 2009). For parsimony of classes, the Lo-Mendell-Rubin adjusted likelihood ratio test with *p* > 0.05 (LRT; Lo et al., 2001) and the Bootstrapped likelihood ratio test with *p* > 0.05 (BLRT; McLachlan & Peel, 2004) were used. Specifically, LMRT and BLRT are significance tests between two different models with *k* classes against *k*-1 classes. The tests with *p* > 0.05 indicate that the *k*-class is better.

The classification diagnostic criteria were assessed using the entropy and average posterior probabilities. Specifically, the entropy statistic was used with values between 0.60 and 0.80 considered as an acceptable range of accuracy (Muthén, 2004; Jung & Wickrama, 2008). Higher entropy values indicate better classification quality (Nagin, 2005). Average posterior probabilities (AvPP) were used to assess the accuracy of a model in classifying individuals into their most likely classes. The average posterior probabilities are presented in a matrix whose diagonals represent the average probability of an individual being assigned to a latent profile, given their scores on the indicator variables used to create the profiles (Muthén & Muthén, 2000). Higher probabilities (close to 1) indicate greater confidence that an individual belongs to that class, while the off-diagonal elements contain the probabilities of cases belonging to one profile being assigned to another profile in the current typology solution. Lower off-diagonal probabilities (closer to 0) are desirable. 0.90 is used as a cut-off for acceptable diagonal probabilities (Muthén & Muthén, 2000).

To enable interpretation and comparison between LPA and cluster analysis (CA), a two-stage clustering procedure was used. In line with previous studies (Gartstein et al., 2017; Liu et al., 2022; Spurk et al., 2020), this approach allows one to understand whether the LPA results are model invariant. First, a hierarchical cluster analysis with Ward method was performed, followed by a *k*-means cluster analysis. The comparison between LPA and CA classification results was performed using the agreement between LPA and CA in terms of assignment of participants to parallel profiles/clusters (overlap). Chi-squared tests were performed to detect agreement between LPA and CA solutions in terms of case assignment (Eshghi et al., 2011; Zani & Cerioli, 2007), and Cramer’s *V* statistics was calculated, with higher values indicating a stronger association between profiles and clusters.

Finally, after confirming the profiles, an R3STEP command for a multinomial logistic regression (Asparounhov & Muthén, 2014) was set in LPA to test whether gender, age (18-24; 25-34), student/employment status, post-covid economic situation and loss episodes during the pandemic predicted profile membership.

## Results

### Identification of Profiles with LPA

Table 1 shows the descriptive analysis (means, standard deviations, skewness, and kurtosis) of all the study variables. As reported, the comparison of the fit indices showed that the three-profile solution was the best selected solution (RQ1). Specifically, the three-profile solution showed the best fit to the data in terms of AIC (13422.153) and BIC (13468.011) and a significant *p*-value of LRT and BLRT (see Table 2). Adding a further class (four-profile solution) did not improve the model fit. Furthermore, the nonsignificant LMR test in the four-profile solution suggests that the more parsimonious model (three-profile solution) is the better fitting and representative model (Ferguson et al., 2019).

**Table 1:** Means, standard deviations, skewness and kurtosis

Variable	*M*	*SD*	*Sk*	*K*
1. FN	3.040	1.038	0.006	*-0.853*
2. FP	3.098	0.904	-0.050	-0.523
3. FC	3.199	1.036	-0.184	-0.777
4. FPers	3.613	0.579	-0.295	0.358
5. FPersp	3.347	0.892	-0.204	-0.389
6. FPlan	3.163	0.633	-0.208	-0.216
7. Dep	20.874	11.842	0.060	-0.994
8. Anx	16.257	11.324	0.399	-0.746
9. Stress	26.802	9.773	-0.463	-0.364
10. PM	18.033	6.608	-0.122	-0.384
11. SM	25.799	6.891	-0.713	0.040
12. LO	17.175	5.198	-0.22	-0.509
13. AQ	2.681	0.608	0.244	-0.452
14. PTF	29.030	5.715	0.207	-0.671

*Note: M* = mean; *SD* = standard deviation; *Sk* = skewness; *K* = kurtosis; FN = Future Negative; FP = Future Positive; FC = Future Confusion; FPers = Future Perseverant; FPersp = Future Perspicuity; FPlan = Future Planning; Dep = Depression; Anx = Anxiety; PM = Presence of Meaning; SM = Search of Meaning; LO = Life Orientation; AQ = Aggressive behavior; PTF = Pandemic, Time and Future.

**Table 2:** Model comparison

Fit statistics	2-Class	3-Class	4-Class
Proportions (%)	55/45	30/29/41	20/27/29/24
AIC	13951.794	13422.153	13217.327
BIC	13985.793	13468.011	13275.045
Entropy	0.899	0.906	0.878
LRT *p* value	< .001	< .001	0.524
BLRT *p* value	< .001	< .001	< .001

*Note*. AIC =Akaike Information Criterion; BIC=Bayesian Information Criterion; LRT = Lo-Mendell-Rubin adjusted likelihood ratio test; BLRT = Bootstrapped likelihood ratio

As seen in Table 3, the classification diagnostic criteria (AvPP and entropy) are satisfactory for the three-profile solution (a value of 0.90 is used as a cut-off for acceptable diagonal probabilities, Muthén & Muthén, 2000). For the three-profile solution posterior probabilities range from 0.953 to 0.973. The three-profile solution does not show a satisfactory level of diagonal probabilities. In addition, a high entropy value (0.906) is shown in the three-profile solution, which is the best for precision in the identification of profiles.

**Table 3:** Classification diagnostics for the best model solutions

Model	AvPP				E
3-profile	0.973	0.000	0.027		0.906
	0.000	9.55	0.045		
	0.024	0.023	0.953		

4-profile	0.911	0.016	0.023	0.050	0.879
	0.029 0.014	0.938 0.000	0.000 0.973	0.034 0.013	
	0.060	0.030	0.023	0.887	

*Note*. AvPP = Average Latent Class Probability; E = Entropy

**Figure 1:**
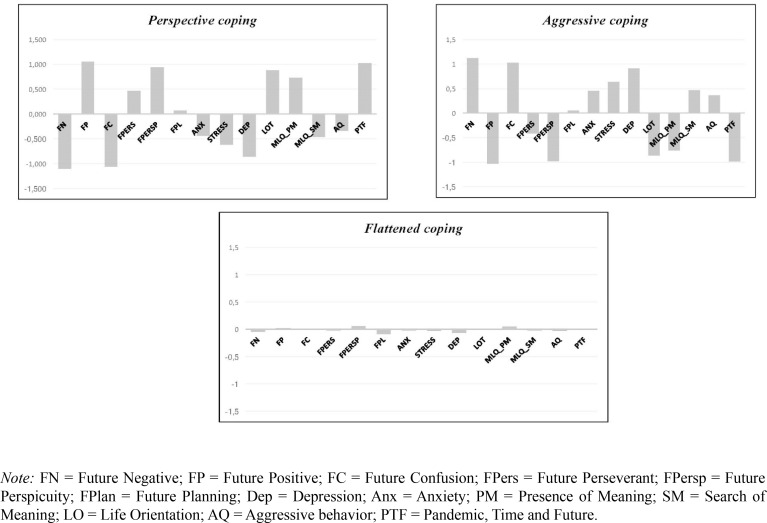
Latent Profiles

Figure 1 shows the three-profile solutions with standardized values on the *y*-axis (mean of 0 and a standard deviation of 1). Profile 1 (30%, *n* = 117) labelled *Aggressive coping* represent individuals with the highest levels of future negative, future confusion, depression, anxiety, stress, search for meaning in life, and aggressive behavior, the lowest level of future positive, future perseverant, future perspicuity, presence of meaning in life, optimism, and situational time perspective, and an average level of future planning. Profile 2 (29%, *n* = 114) is labelled *Perspective coping* as these individuals endorsed the highest level of future positive, future perseverant, future perspicuity, presence of meaning in life, optimism, situational time perspective and future planning and lowest levels of future negative, future confusion, depression, anxiety, stress, a search of meaning in life and aggressive behavior. Profile 3 (41%, *n* = 158) labelled *Flattened coping* since these individuals endorsed the average score across dimensions

### Comparing the LPA solution with cluster analysis (CA)

First, a hierarchical cluster analysis was performed using the Ward method, which links pairs of cases with the smallest distance between them until all cases are linked into one cluster composed of all cases. Fit indices are not available in the case of CA but the usual visual inspection of the dendogram indicated that a three-cluster solution provided the best solution. Clustering results in 3 clusters, as in LPA. The first profile (17%) contains the individuals with the highest levels of future negative, future confusion, depression, anxiety, stress, search for meaning in life, and aggressive behavior, the lowest level of future positive, future perseverant, future perspicuity, presence of meaning in life, optimism, and situational time perspective, and the average level of future planning. The second profile (29%) contains the individuals with the highest level of future positive, future perseverant, future perspicuity, presence of meaning in life, optimism, situational time perspective and future planning and lowest levels of future negative, future confusion, depression, anxiety, stress, a search of meaning in life and aggressive behavior. The third profile (54%) contains the individuals with the average score across dimensions.

Then *k*-means clustering, which is a partitioning method that divides a dataset into *k* distinct and non-overlapping clusters based on similarity, was used to confirm the three-cluster solution. The first profile (30%) contains the individuals with the highest levels of future negative, future confusion, depression, anxiety, stress, search for meaning in life, and aggressive behavior, the lowest level of future positive, future perseverant, future perspicuity, presence of meaning in life, optimism, and situational time perspective, and the average level of future planning. The second profile (34%) contains the individuals with the highest level of future positive, future perseverant, future perspicuity, presence of meaning in life, optimism, situational time perspective and future planning and lowest levels of future negative, future confusion, depression, anxiety, stress, a search of meaning in life and aggressive behavior. The third profile (36%) contains the individuals with the average score across dimensions.

The series of chi-square tests comparing the distribution of cases assigned to matched type based on LPA and CAs classification were significant. In particular, the chi-square test comparing LPA vs hierarchical cluster analysis (Ward method) was significant (*χ^2^* = 406.593, *df* = 4; *p* < .001) with a Cramer’s *V* = 0.723, *p* < .001. The chi-square test comparing LPA vs *k*-means cluster was also significant (*χ^2^* = 442.473, *df* = 4; p < .001) with Cramer’s *V* = 0.754, p < .001. Thus, the LPA and CAs produced relatively comparable profiles/clusters, but each method classified individuals differently, producing a significant chi-square test and different sizes for profiles/clusters.

### Examining the covariates of LPA profiles

Once the profiles were identified, we studied the effect of gender, age group, student/employment status, episodes of loss during the pandemic, and economic situation post-covid on profile belongingness (RQ2). The multinomial logistic regression results, using Profile 1 as the reference group, showed no significant differences for gender, age group, students/employment condition, and episodes of loss during the pandemic. But the economic situation post-covid significantly predicted belonging to Profile 1 (*b* = 0.603, SE = 0.284, *p* = 0.034), showing a higher probability of having this type of profile.

## Discussion

This study was designed to shed light on the personal resources that enables young people to cope with psycho-social challenges. The general aim of the study was to explore the hypothesis that time perspective, optimism and meaning in life are potentially crucial elements in the resolution of young people’s psycho-social challenges and that specifically these psychological dimensions would be connected with each other in defining specific individual modalities of coping with difficult situations (i.e., the covid pandemic).

In order to address this issue, we used a person-centered analysis to identify different types of people in terms of profiles, following four steps: First, we identified profiles using a latent profile analysis (LPA), using time perspective, meaning in life, optimism, and well-being/malaise variables. Second, we explored the characteristics of the subjects belonging to each group in terms of gender, age (18-24; 25-34), students/employment condition, economic situation post-covid and episodes of loss during the pandemic multinomial. And third, we described the overall empirical profiles that emerged.

Our findings reveal a relationship between profiles of time perspective, meaning in life, and optimism, on the one hand, and negative psychosocial functioning (anxiety, depression, aggressive behavior) on the other. In addition, the results confirm the hypothesis that profiles with the highest levels of future orientation in coping with problematic situations, and of optimism and meaning in life have the lowest levels of negative psychological functioning.

In our results, we identified three profiles describing different patterns of time perspective, meaning in life, and optimism and negative psychosocial functioning, that we have defined in terms of different “perspective coping”. To elaborate them, in terms of well-being and internalizing symptoms we found one “positive” profile (perspective coping), one “negative” profile (aggressive coping) and one “ambivalent” profile (flattened coping).

The positive profile (Profile 2; Perspective coping) identified a large group of young people who have a positive orientation to the future, supported by optimism and oriented by the presence of meaning in life. As for the negative psycho-social dimensions, these young people have the lowest scores on these variables. Thus, this profile suggests that to have a clear and hopeful vision of the future, when embodied by a clear meaning in life, is a positive resource for coping with difficult situations. In this sense, it could serve as a protection factor. We therefore interpreted this profile by describing it as a prospective coping modality: it is having a vision of one's future, accompanied by self-clarity and understanding of one’s life project which constitutes a protective factor for individual psychological well-being and has allowed young people not to get disoriented in a moment of specific and unpredictable difficulty.

The negative profile (Profile 1; Aggressive coping) identified a smaller group of young people in the study. This profile describes individuals with the highest levels of future negative, future confusion, depression, anxiety, stress, search for meaning in life, and aggressive behavior. In this case, young people show difficulty in coping with the problematic situation. Their coping is characterized by depressive dimensions, an overall sense of disorientation and discouragement for the future. A sense of confusion and anxiety is associated with fear for the future and above all with high aggression scores. In this case, it is defined as a problematic, externalizing and disoriented way of dealing with difficulties. In this case, the development task that took place during the Covid period can serve as an example of the difficulty this group of young people have in orienting themselves in the future when they have not understood the meaning of their lives. This cluster shows us, negatively, how important it is to have direction and meaning in one’s life, as studies on the topic indicate (Steger et al., 2009). This is particularly true for young people who find themselves immersed in a moment of their lives in which they need to grapple with planning and building their own future to define their own identity (Sica et al., 2016). Indeed, satisfactory levels of meaning in life allow individuals to find themselves in a balanced condition of overall well-being and adjustment (Schnell, 2009).

The third profile describes individuals with average scores across all dimensions. We have defined this profile as a Flattened profile, that is attenuated in the sense that the young people composing it are not polarized towards high or low scores but remain within a range of average scores on all dimensions examined. This can be interpreted as the tendency of these young people to face problematic situations with an overall involvement of negative and positive experiences, but also with an undefined orientation towards the future. Even more stable dimensions, such as optimism, seem to only partially characterize these young people. This profile, the most numerous among the three that emerged, probably represents an overall “indifferentiation” which reflects that characteristic of young people, also found in studies on identity definition processes (Sestito et al., 2015), according to which young people are still undefined and have not built a project for themselves, remaining somehow suspended, but also open to possible directions. Precisely this “openness” represents, from an application point of view, a flexible element on which to intervene to stimulate young people towards a greater acquisition of meaning, planning, persistence and resourcefulness; that is, towards a positive developmental trajectory.

In addition to the specificities defined by the profiles, our results also showed an overall transversality of the dimensions considered both by gender and by age. It is therefore not these elements that can discriminate the ways of dealing with problematic situations, but rather their specific mutual relationship. In detail, no differences linked to the two age groups emerged, suggesting that the psychological processes of orientation to the future, as well as personal psychological resources, do not undergo substantial changes in the transition from late adolescence to early adulthood. This evidence helps us to think that supporting and strengthening individual resources is, from a positive psychological perspective, a valid strategy at every age stage. On the other hand, it also helps us to believe that, precisely by virtue of their relative stability, such interventions should be carried out as early as possible to support positive and stable development trajectories.

Overall, our results also confirm how meaning in life, optimism and a positive vision of the future represent those dimensions of flourishing (Seligman, 2011) that help in positive psychological development (Gable & Haidt, 2005).

### Limitations and Suggestions for Future Research

Before discussing the implications, we should note that this study has a number of limitations that need to be considered in future research. Firstly, the study focuses on one group of late adolescents, and longitudinal research is therefore needed to support a more specific set of conclusions around identity development. In addition, these are cross-sectional data which prevent us from drawing causal conclusions. Secondly, all the measures used were self-reported, and therefore the data may be influenced by a reporting bias (acquiescence, positivity bias, social desirability). Furthermore, the nature of the explored constructs, positively connoted, could affect the results, as all of them capture some aspects of positive functioning. Thirdly, this study used convenience sampling. Convenience sampling is a non-probability sampling strategy in which participants are recruited on the basis of their accessibility. Unlike results from a random sample, convenience sampling produces estimates that lack generalizability, may have insufficient power to detect differences between sociodemographic subgroups, and contain noise due to sociodemographic variation that cannot be controlled for or accounted for (Bornstein et al., 2023). Therefore, although the present study benefits from a relatively large sample of Italian university students, this sample is not representative (and therefore not generalizable) of Italian university students. Moreover, the perspective coping proposal is based on Italian data and therefore requires a cross-cultural comparison to be generalized. Finally, the study was conducted during the covid-19 pandemic and it would be interesting to replicate the data collection in a post-pandemic period to compare profile distribution checking typologies and characteristics.

In order to remedy the aforementioned limitations, future research could use: a mixed approach to data collection (quantitative and qualitative) (Seginer, 2009; Sica, 2009); a longitudinal design to grasp changing or stabilization of the profiles during the life course; and a cross-cultural perspective to validate the perspective coping as culture free resources for positive psycho-social development.

### Study Implications and Take-Home Message

As noted in the discussion, our findings have important theoretical and practical implications. Firstly, the research fits into the strand of studies that investigate the interplay between positive resources, meaning in life and well-being in adolescents and young adults by increasing knowledge. The use of latent profile analysis allows a typology of protective health resources and the identification of the most vulnerable profiles. In particular, it is possible to plan different interventions on the basis of the profile typification shown: working on the personal direction and meaning of life seems to be crucial in order to combat the difficulties faced by adolescents. Highest levels of internalizing problems, such as anxiety and depression, and externalizing problems, such as aggression, are indeed found in individuals who show anxiety about the future and low levels of meaningfulness. Working on a clear and hopeful vision of the future and a sense of meaning in life means focusing on those positive resources that are useful in coping with difficult situations.

### Compliance with Ethical Standards

The authors declare that the current research was conducted according to ethical standards. Before undertaking the study, permission to administer anonymous self-report questionnaires was obtained from the principals of the high schools. Informed consent was used with all participants and for adolescents below the age of 18, parental consent was obtained. Participation in the study was voluntary and anonymity was guaranteed. The ethical implications of the current research were consistent with the Ethical Code of the University of Naples Federico II (prot. no. 12/2021, April 26, 2021).

## Data Availability

The research data analyzed in this paper are available from the corresponding author on request.

## Action editor

Lars-Gunnar Lundh served as action editor for this article.

## Open access

This article is distributed under the terms of the Creative Commons Attribution 4.0 International License (http://creativecommons.org/licenses/by/4.0/), which permits unrestricted use, distribution, and reproduction in any medium, provided you give appropriate credit to the original author(s) and the source, provide a link to the Creative Commons license, and indicate if changes were made.
